# Normal kidney size and its influencing factors - a 64-slice MDCT study of 1.040 asymptomatic patients

**DOI:** 10.1186/1471-2490-9-19

**Published:** 2009-12-23

**Authors:** Bernhard Glodny, Verena Unterholzner, Bernadette Taferner, Karin J Hofmann, Peter Rehder, Alexander Strasak, Johannes Petersen

**Affiliations:** 1 Department of Radiology, Innsbruck Medical University, Anichstrasse 35, 6020 Innsbruck, Austria; 2 Neurourology, Department of Neurology, Innsbruck Medical University, Innsbruck, Austria; 3 Innsbruck Medical University, Department of Medical Statistics, Computer Sciences and Health Management, Innsbruck, Austria

## Abstract

**Background:**

Normal ultrasound values for pole-to-pole kidney length (LPP) are well established for children, but very little is known about normal kidney size and its influencing factors in adults. The objectives of this study were thus to establish normal CT values for kidney dimensions from a group of unselected patients, identify potential influencing factors, and to estimate their significance.

**Methods:**

In multiphase thin-slice MDCTs of 2.068 kidneys in 1.040 adults, the kidney length pole to pole (LPP), parenchymal (PW) and cortical width (CW), position and rotation status of the kidneys, number of renal arteries, pyelon width and possible influencing factors that can be visualized, were recorded from a volume data set. For length measurements, axes were adjusted individually in double oblique planes using a 3D-software. Analyses of distribution, T-tests, ANOVA, correlation and multivariate regression analyses were performed.

**Results:**

LPP was 108.5 ± 12.2 mm for the right, and 111.3 ± 12.6 mm for the left kidney (p < 0.0001 each). PW on the right side was 15.4 ± 2.8 mm, slightly less than 15.9 ± 2.7 mm on the left side (p < 0.0001), the CW was the same (6.6 ± 1.9 mm). The most significant independent predictors for LPP, CW, and PW were body size, BMI, age, and gender (p < 0.001 each). In men, the LPP increases up to the fifth decade of life (p < 0.01). It is also influenced by the position of the kidneys, stenoses and number of renal arteries (SRA/NRA), infarctions suffered, parapelvic cysts, and absence of the contralateral kidney; CW is influenced by age, position, parapelvic cysts, NRA and SRA, and the PW is influenced in addition by rotation status (p < 0.05 each). Depending on the most important factors, gender-specific normal values were indicated for these dimensions, the length and width in cross section, width of the renal pelvis, and parenchyma-renal pyelon ratio.

**Conclusions:**

Due to the complex influences on kidney size, assessment should be made individually. The most important influencing factors are BMI, height, gender, age, position of the kidneys, stenoses and number of renal arteries.

## Background

Normal ultrasound values for pole-to-pole kidney length (LPP) are well established for children [1,2], adults [3,4], and seniors [5]. A short LPP usually allows chronic kidney failure to be easily distinguished from acute kidney failure with normal or enlarged values [6,7]. Moreover, changes in LPP, parenchymal width (PW), cortex width (CW), or volume can be associated with atherosclerotic renal disease [8], arterial hypertension [9], atherosclerotic renovascular disease [10], or diabetes mellitus [11], or be indicative of these. The renal dimensions also allow conclusions as to the single kidney glomerular filtration rate to be made [12]. Aside from the acceptable reliable estimation of the LPP by ultrasound [9], all dimensions can now be easily determined by MRI [10], CT [8], and probably ultrasound as well. However, thus far no normal values exist, and the few known LPP values of healthy persons [8,10] are different from the ones indicated by ultrasound [4]. The objectives of this study were thus to establish normal CT values for kidney dimensions from a group of unselected patients and identify potential influencing factors.

## Methods

### Patients

A total of 1,040 consecutive patients were included in this retrospective cross-sectional, observational CT study. The average age of the 456 women and 583 men (male/female ratio 1:1.28) was 60 ± 15.7 years (range: 19 - 99.4 years). For 27.7% of the patients, the indication for the CT was a tumor, for 21.1% vascular pathology, for 20.5% pathologies of the liver, for 2.9% pathologies of the bile ducts, for 3.2% bleeding, and for 24.6% miscellaneous reasons. The study was conducted subject to the guidelines of the Declaration of Helsinki. It had no influence on the treatment of the patients, and no influence on the execution or indication of the CT's. Institutional ethical approval was not necessary, as the study had no influence on treatment. The institutional ethical review board did not require its approval for this retrospective cross-sectional study.

### Imaging

All examinations were conducted on the same 64-slice MDCT scanner (Lightspeed VCT XT, General Electric, Milwaukee, USA). Some 41.6% of the patients were examined with liver, 35.8% with angiography, and 12.6% with pancreas protocols. After a standardized weight-based administration of Iodixanol 320 with a flow of 5 ml/s (body weight in kg × 2 = contrast agent in ml; Visipaque, GE Healthcare, Cork, Ireland), a bolus tracking procedure (SmartPrep, General Electric, Milwaukee, USA) was used to obtain a scan of the upper abdomen in an early arterial phase 2.5 mm slice thickness (ST), tube voltage (TV) 120 kV, tube current modulation (TCM), noise index (NI) 16, scan field of view (SFOV) ~50, pitch 0.984:1), of the abdomen in a portal venous phase (5 mm ST; TV 120 kV, TCM, NI 18, SFOV 50, pitch 0.984:1), and of the abdomen 200 seconds later (2.5 mm ST). The aortic protocol included imaging the aorta in 0.625 or 1.25 mm thick slices (TV 120 kV, TCM, NI 40, SFOV 50, pitch 0.984:1), and a subsequent scan of the abdomen in a venous phase. The pancreas protocol was similar to the first, modified by a delay of 3 seconds before the arterial phase and omitting the fourth phase. Other protocols were used for 11% of the patients. Coronal and sagittal or axial reformatted images were prepared using a standard convolution kernel. The 0.625 mm collimated source images were saved temporarily on a workstation from General Electric (AW 4.4, GE, Milwaukee, USA).

### Inclusion and exclusion criteria

Inclusion criteria were contrast in the aorta of >200 HU in the arterial phase and the existence of a venous contrast medium phase with collimation of 0.625 mm. Not reaching the threshold (n = 468), an incomplete image of the urogenital tract, movement artifacts, or technical defects (n = 18), history of known kidney or lower urinary tract disease or a related indication for the examination (n = 31), diseases of the renal parenchyma visible in CT (n = 11), and age under 18 years (n = 32) were grounds for exclusion. No patients with a creatinine level >2 mg/dl were examined.

### Execution of the study

Taking all preliminary examinations (n = 4.580 abdominal CTs, 4.4 ± 4.3 examinations/patient; range: 0-30) into consideration, image analysis was carried out by consensus of two radiologists. There was at least one preliminary examination available for 949 patients. The software used was 3D PACS (Tiani 3D PACS software, version 3.3.16, Agfa-Gevaert N. V., Mortsel, Belgium), with which individual reconstructions were also carried out. The study design was retrospective cross-sectional. If, after verification of the in- and exclusion criteria, an examination could be included into the study, the 0.625 mm collimated source images, which had been temporarily saved on the workstation, were sent to the Picture Acquisition and Communication System (PACS). This procedure was performed retrospectively, once a week. All image analyses were carried out using a diagnostic monitor (Lenovo 6659 HG2, IBM, Raleigh, Morrisville, NC, USA), in consensus of two experienced radiologists.

### Contrasting the vessels

In the 1.040 CTs, contrasting of the vena cava inferior cranial to the renal veins was 99.9 ± 55.8 HU, of the aorta in the arterial phase at the level of the renal arteries 301.6 ± 76.9 HU.

### Parameters measured

**Figure 1:**
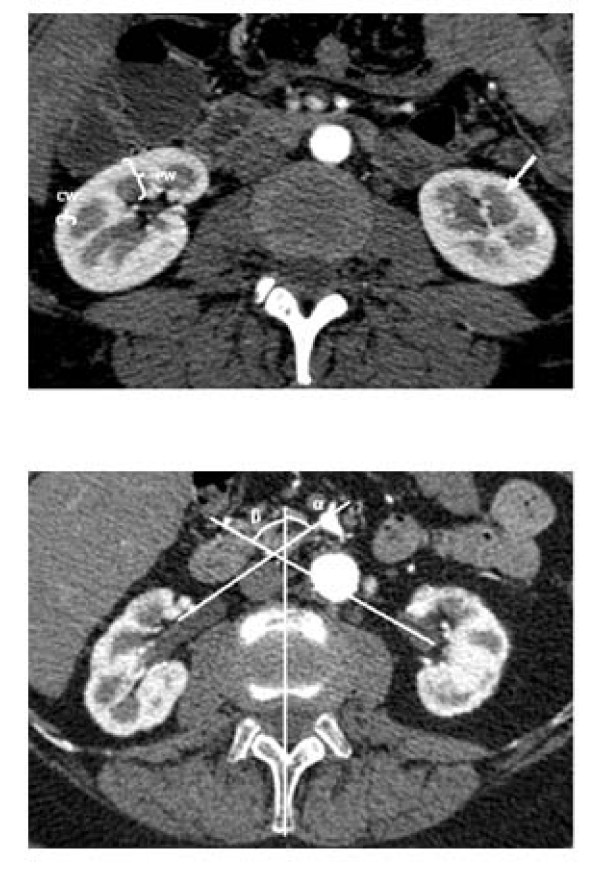
Axial 0.625 mm collimated slice of the kidney in an arterial phase, with the strongly contrasted kidney cortex (*), and a renal pyramid (arrow). Cortical width (CW), and parenchymal width (PW) (a). Axial 0.625 mm collimated slice of the kidney in an arterial phase, with depiction of the kidney pelvis, and the rotation status of the kidney pelves in relation to the reference sagittal median plane (b). Pelvic angle on the right side (α), and on the left side (β).

The parameters measured were the number of kidneys, LPP, kidney length (KL) and width (KW) in axial slices, width of the parenchyma (PW) and the cortex (CW) in the arterial phase (Figure 1a and 1b), the width of the renal pelvis (WRP), ratio of the parenchyma to the renal pyelon (RPRP), the rotation status of the renal pelvis measured in the axial plane in relation to the reference sagittal median plane (AR), and the rotation status of the kidneys as measured in the sagittal plane in relation to the reference coronal plane (SR). As a control of quality, the measurements were performed twice in a random sample of 50 data sets. The intraclass correlation coefficient showed a very high reliability of the data, with values between 0.96 (cortical width) and 0.99 (kidney length). The number of additional (ADRAs) or accessory renal arteries (ACRAs) from the aorta or its branches was determined as defined by Satyapal [13], as well as the number of veins. Influencing factors considered were age, gender, height and BMI, position of the midpoints of the renal pelvis (RP, L1 = 1, L2 = 2 etc.) and the upper poles (UPP) in relation to the spine, minimal vertical length from the surface of the kidney to the dorsal fascia (ventral position, VP), width of the renal pelvis, qualitative stenoses of the renal arteries, infarctions, parapelvic cysts, and concrements. Incomplete and complete double ureters, but not bifid pelvis, were assessed as duplex systems.

### Statistics

All values are given as mean value ± standard deviation in the normal value tables with the median the 2^nd^, 10^th^, 90^th ^and 98^th ^percentiles. Descriptive statistics were compiled using the Excel program (Microsoft, Seattle, Washington, USA). Kolmogoroff-Smirnov tests were used for distribution analyses of the data. Correlation analyses have been performed using the method of spearman. Group comparisons were made using parametric and nonparametric two-tailed t-tests where appropriate and indicated (Whitney-Mann Test, WMA). Comparisons of several groups were made using one-way analysis of variance (one-way ANOVA) with post hoc Dunn tests (GraphPad Prism version 4.00, GraphPad Software, San Diego. California USA). To identify influencing factors on the target variables LPP, CW, and PW, multivariate regression models were adapted using a forward stepwise selection procedure (SPSS 15, SPSS Inc., Chicago, Illinois, USA). The same variables on the contralateral side, as the strongest predictors, were excluded from the models. A p < 0.05 was considered statistically significant.

## Results

### Anatomical peculiarities

There were 1.033 kidneys on the right side, compared with 1.035 on the left. Six kidneys on the right, and 5 kidneys on the left had been removed. In one patient, there was a right sided kidney agenesis. There were 24 (2.3%) right duplex systems and 20 (1.9%) left duplex systems. Of these, 9 (0.87%) were complete on the right, and 7 (0.68%) on the left side. In 253 patients, there were 277 ADRAs and 6 ACRAs on the right and in 265 patients, 291 ADRAs and 6 ACRAs on the left. There were thus 1.316 renal arteries from the aorta or its branches on the right and 1.332 on the left.

### Dimensions and position of the kidneys-normal kidney size

Table 1 shows the normal values for the parameters measured, and Table 2 the normal values for kidney size, split into groups according to female and male gender, and according to the right and the left side. Kidneys with any pathologic conditions, like renal artery stenoses, have been excluded in these tables, as well as single kidneys. For comparison between the sides, the Wilcoxon matched pairs test was used, because the left, and the right kidney form a pair.

**Table 1:** Normal values for kidney length pole to pole, cortical and parenchymal width, classified according to the side of the kidney

	Side	Mean	Median	SD	p (Wilcoxon test)
**Kidney length**	right	108.5	108	12.2	<0.0001
	left	111.3	111	12.6	

**Parenchymal width**	right	15.4	15.4	2.8	<0.0001
	left	15.8	15.8	2.7	

**Cortical width**	right	6.6	6.5	1.9	>0.05 (ns)
	left	6.6	6.5	2.0	

**Width (axial planes)**	right	51.3	50.8	7.8	<0.0001
	left	53.3	52.9	8.2	

**Length (axial planes)**	right	57.7	57.4	8.0	<0.0001
	left	53.6	52.9	8.2	

**Width of the pyelon**	right	18.5	17.6	6.2	<0.0001
	left	19.9	19.1	6.3	

**Position of the midpoint of the renal pelvis**	right	2.2	2.2	0.7	<0.0001
	left	2.1	2.1	0.6	

**Position of the upper pole of the right kidney**	right	0.6	0.6	0.8	<0.0001
	left	0.4	0.5	0.9	

**Rotation status in sagittal planes (upper pole versus lower pole in relation to a reference coronal plane)**	right	25.8	25.3	11.1	>0.05 (ns)
	left	24.3	11.0	23.8	

**Rotation status of the renal pelvis in the axial plane, in relation to the reference sagittal median plane**	right	60.3	58.4	18.1	<0.0001
	left	53.5	49.0	22.5	

**Parenchym/pyelon ratio**	right	0.9	0.9	0.4	>0.05 (ns)
	left	0.9	0.8	0.5	

**Table 2:** Normal values for kidney length pole to pole, cortical and parenchymal width, classified according to the side of the kidney, and gender

		Mean	Median	95% Confidence interval of mean		SD	2% Percentile	10% Percentile	90% Percentile	98% Percentile	Wilcoxon test
		
		(mm)	(mm)	Lower	Upper	(mm)	(mm)	(mm)	(mm)	(mm)	p value
**Women**	**Kidney length right side**	103.8	104	102.8	104.9	11.1	79.6	90.31	117.9	128.0	<0.0001
	**Kidney length left side**	106.3	106	105.3	107.4	11.5	82.5	92.12	121.9	130.9	
	**Parenchymal width right**	14.5	14.4	14.3	14.7	2.6	8.9	14.26	14.74	20.4	<0.0001
	**Parenchymal width left**	15	14.8	14.7	15.2	2.4	10.7	12.3	18.4	20.2	
	**Cortical width right side**	6.2	6.1	6.1	6.3	1.3	3.8	4.6	8.2	9.4	ns
	**Cortical width left side**	6.2	6.2	6.1	6.3	1.2	3.7	4.7	7.7	8.6	
	**Width right (axial planes)**	48	47.2	47.3	48.6	7.2	34.9	40.2	57.6	65.2	<0.0001
	**Width left (axial planes)**	49.4	49.3	48.7	50.1	7.4	35.8	40.6	58.1	67.6	
	**Length right (axial planes)**	55.8	55.8	55	56.6	7.9	39.5	46.8	64.6	73.9	<0.0001
	**Length left (axial planes)**	51.2	50.8	50.4	52.1	8.3	35.6	42	61.9	71.5	
	**Width of the pyelon (right side)**	16.9	16.1	16.4	17.4	5.7	7.5	10.3	11.5	31.3	<0.0001
	**Width of the pyelon (left side)**	18.0	17.1	17.5	18.5	5.6	9.1	24.6	26.0	30.9	
	**Parenchym/pyelon ratio (right side)**	1.0	0.9	0.9	1.0	0.4	0.4	0.6	1.4	1.8	= 0.0215
	**Parenchym/pyelon ratio (left side)**	0.9	0.9	0.9	1.0	0.6	0.4	0.5	1.3	1.8	

**Men**	**Kidney length right side**	112.0	112.0	111.1	113.0	11.6	87.8	99.0	126.0	136.4	<0.0001
	**Kidney length left side**	114.9	115.0	113.9	115.9	12.0	88.0	100.0	130.0	140.0	
	**Parenchymal width right**	16.3	16.1	15.9	16.4	2.7	10.4	12.9	19.5	21.8	= 0.0002
	**Parenchymal width left**	16.5	16.4	16.3	16.7	2.4	11.3	13.2	19.8	22.3	
	**Cortical width right side**	6.8	6.7	6.7	7.0	2.2	3.9	5.0	8.6	10.2	= 0.0470
	**Cortical width left side**	7.0	6.9	6.8	7.2	2.7	4.3	5.0	8.8	10.1	
	**Width right (axial planes)**	53.9	53.4	53.3	54.5	7.2	39.7	45.4	63.0	70.2	<0.0001
	**Width left (axial planes)**	56.4	56.3	55.8	57.4	7.5	42.1	47.4	65.6	73.1	
	**Length right (axial planes)**	59.3	58.9	58.5	60.0	7.9	44.5	50.0	69.0	78.0	<0.0001
	**Length left (axial planes)**	55.5	54.5	54.8	56.3	8.0	39.6	45.9	66.5	74.4	
	**Width of the pyelon (right side)**	19.7	18.7	19.1	20.2	19.7	8.3	12.5	28.0	35.06	<0.0001
	**Width of the pyelon (left side)**	21.5	20.7	20.9	22.0	21.5	9.6	13.8	30.7	35.1	
	**Parenchym/pyelon ratio (right side)**	0.9	0.8	0.9	1.0	0.5	0.4	0.5	1.4	2.0	<0.0001
	**Parenchym/pyelon ratio (left side)**	0.9	0.8	0.8	0.9	0.4	0.4	0.5	1.3	1.8	

### Kidney size in relation to age, height, and BMI

**Figure 2:**
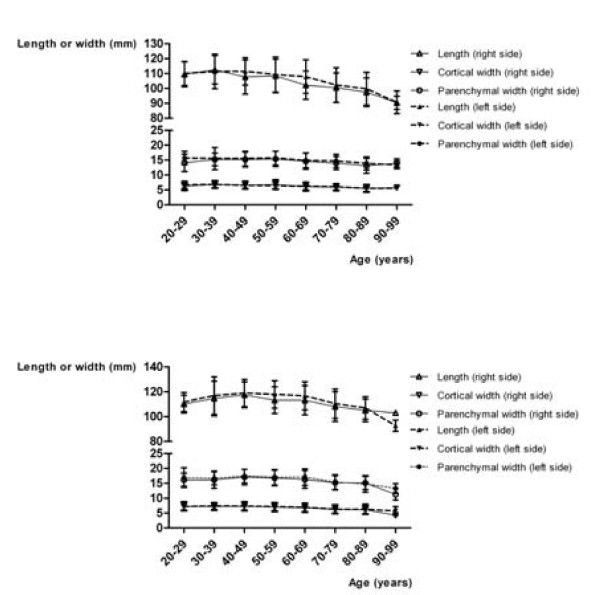
**Kidney length (pole to pole) in oblique sagittal planes, and cortical and parenchmal width in axial planes, classified according to age, gender and the side of the kidney**. Women (a), and men (b).

**Figure 3:**
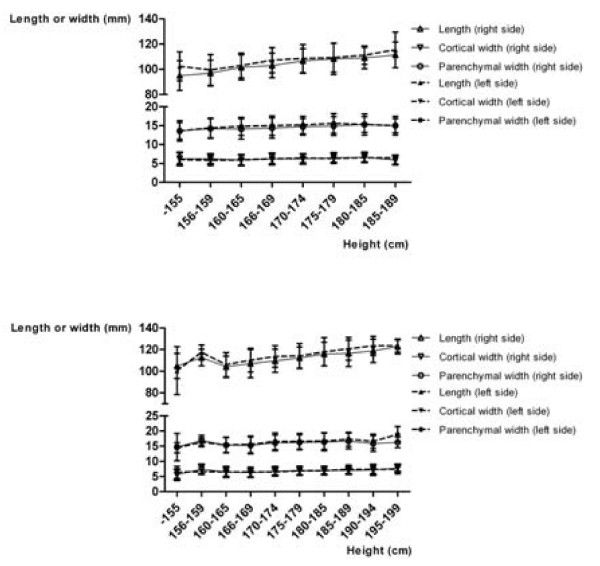
**Kidney length (pole to pole) in oblique sagittal planes, cortical and parenchmal width in axial planes, classified according to height, gender and the side of the kidney**. Women (a), and men (b).

**Figure 4:**
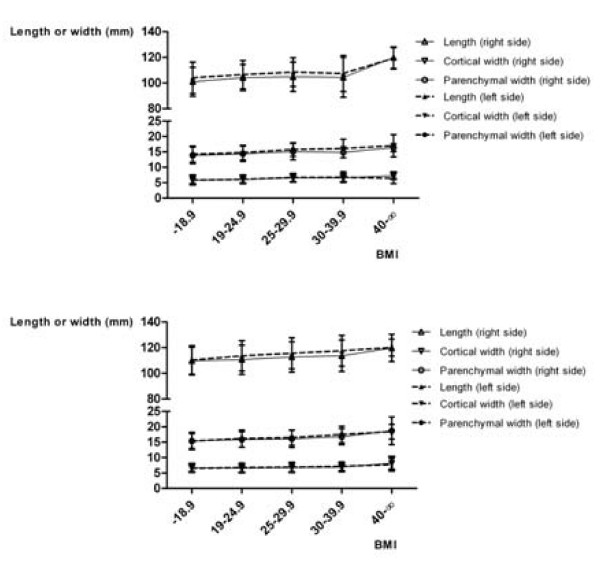
**Kidney length (pole to pole) in oblique sagittal planes, cortical and parenchmal width in axial planes, classified according to BMI, gender and the side of the kidney**. Women (a), and men (b).

Figure 2 shows the mean values for the LPP, CW, and PW in relation to gender and age. In men, the LPP increases up to the fifth decade (p = 0.0053 on the right; p = 0.0012 on the left, ANOVA), the PW also to a slight extent (p = 0.0293 on the right, p = 0.2924 on the left, ANOVA). The CW remains the same in this period. Beginning in the fifth decade, the sizes decrease in both genders (p < 0.0001 each). Figure 3 shows the mean values of LPP, CW, and PW in relation to gender and height. There are linear correlations between height and LPP, CW, and PW, which are statistically significant (p < 0.05 each, Spearman ρ's between 0.13 and 0.4). Figure 4 shows the mean values of LPP, CW, and PW in relation to gender and BMI. There are linear correlations between the BMI and LPP, CW, and PW. Except for the PW in men, they are statistically significant (p < 0.05 each, Spearman ρ's between 0.13 and 0.24).

### Parenchyma-renal pyelon ratio

**Figure 5:**
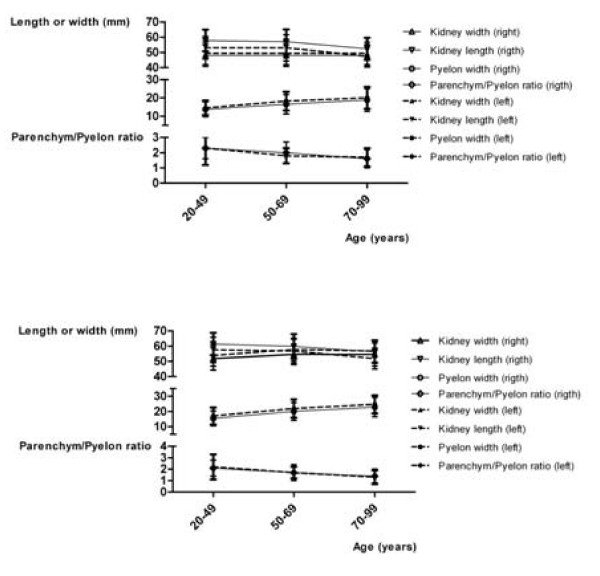
**Kidney width and length in axial planes, width of the pyelon, and parenchym/pyelon ratio, classified according to age, gender and the side of the kidney**. Women (a), and men (b).

The RPRP is inversely related to age (Figure 5) and BMI, for the latter with correlation coefficients of ρ = -0.1010 (Spearman, p = 0.0394) on the right and ρ = -0.0786 on the left for women, (Spearman, p = 0.1090), and for men ρ = -0.1993 on the right (Spearman, p < 0.0001) and ρ = -0.1198 on the left (Spearman, p = 0.0061).

### Kidney width and length in axial slices

In men there is a weak correlation between KW and age (Figure 5), but no correlation between KW and height (p > 0.05 each). The KW is correlated to the BMI with Spearman ρ values of 0.2802 on the right and 0.2958 on the left in women, 0.3873 on the right and 0.3525 on the left in men (p < 0.0001 each). The KL decreases with age (Figure 5), but increases with the BMI. Correlation coefficients for women are 0.1106 (p = 0.0383) on the right and 0.1123 (p = 0.0352) on the left, for men 0.0905 (p = 0.0566) on the right and 0.1030 (p = 0.0300) on the left. The KL is correlated to height with Spearman ρ values for women of 0.2985 (p < 0.0001) on the right and 0.1994 (p = 0.0002) on the left, for men 0.1346 (p = 0.0045) on the right and 0.1493 (p = 0.0016) on the left.

### Sizes in the absence of a contralateral kidney

The 12 single kidneys, at 127 ± 12.7 mm, were longer (p < 0.0001, WMT) than the kidneys in patients with two kidneys. The CW and PW were 8.6 ± 1.8 mm (p = 0.0004 WMT) and 19.5 ± 2.8 mm respectively (p < 0.0001, WMT), wider than the average.

### Sizes of duplex systems

Unilateral duplex kidneys (n = 32) with an LPP of 115.1 (116) ± 14.6 mm compared to 109.4 (107) ± 11.3 mm on the contralateral side, were longer (p = 0.0254) than the contralateral kidneys. Duplex kidneys (n = 44) in general had an LPP of 116.8 (119) ± 13.4 mm, longer than the other kidneys (p = 0.0003). Parenchyma and cortex showed no differences (p > 0.05).

### Sizes of kidneys with several arteries

**Figure 6:**
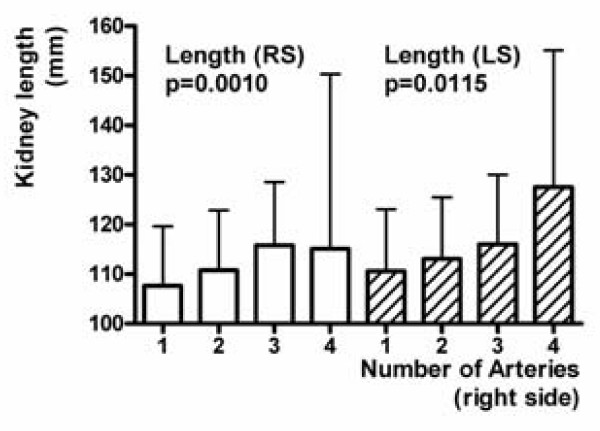
**Kidney length pole to pole (LPP) on both sides in relation to the number of right ADRAs and ACRAs**.

**Figure 7:**
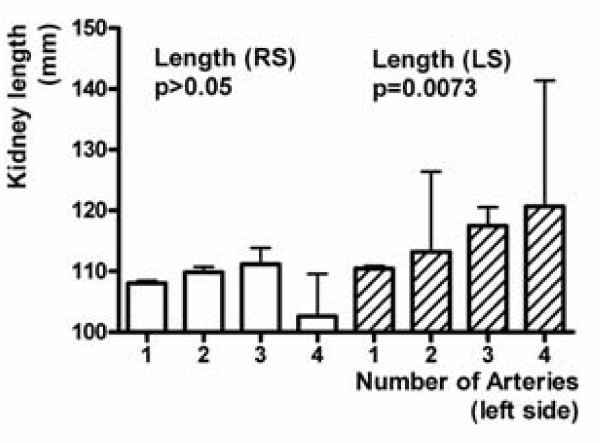
**Kidney length pole to pole (LPP) on both sides in relation to the number of left ADRAs and ACRAs**.

The LPP increases on both sides in relation to the number of right ADRAs and ACRAs (Figure 6; p = 0.0010, and p < 0.0001). The LPP on the left side also increases depending on the number of left ADRAs and ACRAs (Figure 7; p = 0.0073).

### Factors influencing cortical width, parenchymal width, and kidney length

The independent predictors of the CW, PW, and LPP are shown in Table 3. Forward stepwise selection procedures were applied. Gender, BMI, and absence of the contralateral kidney were represented in each of the six models as factors influencing the CP, PW, and LPP.

**Table 3:** The different influencing factors on kidney length pole to pole, cortical and parenchymal width in multivariate regression models

Right side	Factor	β	P value	Step of entry
**Kidney length (pole to pole)**	**Height**	0.35	<0.001	1
	**BMI**	0.22	<0.001	2
	**Stenosis of renal arteries**	-0.15	<0.001	3
	**Male gender**	0.2	<0.001	4
	**Cranial position**	0.16	<0.001	5
	**Ventral position**	-0.157	<0.001	6
	**Absence of left kidney**	0.102	<0.001	7
	**Parapelvic cysts**	0.09	= 0.001	8

**Cortical width**	**Male gender**	0.123	<0.002	3
	**Age**	-0.114	= 0.001	2
	**BMI**	0.105	= 0.002	1
	**Caudal position**	-0.084	= 0.019	7
	**Stenosis of renal arteries**	-0.074	= 0.024	5
	**Absence of left kidney**	0.71	= 0.021	4
	**Parapelvic cysts**	-0.071	= 0.022	6
	**Number of renal arteries**	0.069	= 0.026	8

**Parenchymal width**	**Male gender**	0.22	<0.001	1
	**BMI**	0.15	<0.001	3
	**Height**	0.12	<0.001	2
	**Ventral position**	-0.1	= 0.005	4
	**Parapelvic cysts**	-0.1	<0.001	5
	**Caudal position**	-0.09	= 0.005	7
	**Absence of the left kidney**	0.084	= 0.004	6
	**Age**	-0.73	= 0.026	8

**Left side**	**Factor**	**β**	**P value**	**Step of entry**

**Kidney length (pole to pole)**	**Height**	0.34	<0.001	1
	**BMI**	0.19	<0.001	2
	**Male gender**	0.17	<0.001	5
	**Ventral position**	-0.14	<0.001	6
	**Cranial position**	0.13	<0.001	7
	**Absence of right kidney**	0.1	<0.001	4
	**Number of renal arteries**	0.1	<0.001	3
	**Stenoses of renal arteries**	-0.07	= 0.01	8
	**Previous infarction**	-0.07	= 0.014	9

**Cortical width**	**BMI**	0.152	<0.001	3
	**Age**	-0.151	<0.001	2
	**Male gender**	0.142	<0.001	1
	**Absence of the right kidney**	0.079	= 0.012	4

**Parenchymal width**	**BMI**	0.19	<0.001	1
	**Height**	0.17	<0.001	2
	**Male gender**	0.14	<0.001	3
	**Age**	-0.14	<0.001	4
	**Absence of the right kidney**	0.11	<0.001	5
	**Stenosis of a renal artery**	-0.09	= 0.003	6
	**Constriction of the left renal vein in the aortomesenteric angle**	-0.08	= 0.012	7

## Discussion

Although ultrasound is the primary method of choice for examining the kidneys, we preferred spiral CT for this study because on the one hand, some parameters were not measurable by ultrasound, and on the other hand, individual reformatting in all dimensions with resolutions up to the sub-millimeter range could be made from the volume data sets retrospectively, allowing to anticipate exceedingly precise measurements. Depending on the contrast medium phase, it is possible to delineate the renal structures and collecting system exactly and assess the surrounding organs precisely. Some of the influencing factors indicated for the LPP were already known, others, such as the number of vessels or position of the kidney were not, although their effect is sometimes pronounced and certainly should be taken into account when assessing size in individual cases.

The values for LPP correspond very closely with those indicated for ultrasound [4]. In addition to the assessment of coronal reformatting, the most reliable method of determining the LPP [14], sagittal and individual reformatting were also used, depending on the rotation of the kidneys, so that we must assume the greatest possible reliability of the results. The thin slice, multidetector technique has been chosen in order to obtain very accurate data.

Yet we must point out a few weaknesses of the study.

The patient group was not a randomly selected sample. The alternative, selecting a real random sample, was not possible due to the danger from radiation exposure from the CT. The extent of a potential bias effect, if any exists, should be minimized by the large number of patients with various illnesses unrelated to the kidneys and lower urinary tract. In order to estimate the extent of their influence, diseases of the kidney and lower urinary tract apparent in the imaging were not excluded if they were not known or symptomatic in the patient history and did not affect the renal parenchyma. Omitting the data from these patients did not lead to a change in the kidney sizes in the area indicated. Due to the exclusion of clinically conspicuous "maximum variants" of these diseases we can assume only that the influences subordinate to the main factors were somewhat underestimated. Patients in the initial stages of chronic kidney disease might have been included in the patient group accidentally; however, they were probably under-represented due to negative selection by the choice of the scanner in comparison to a completely randomly selected sample. After a diagnosis was made, each CT was evaluated only once during the study, and not, as would have been desirable, twice by two different observers. To minimize errors, this evaluation was carried out by two observers in consensus. The important issue of transferability of the values to ultrasound cannot be addressed for most of the measurements, but the almost exact correspondence of the LPP values with ultrasound data [4] can be considered a good indication of transferability.

The main advantage of a MD-CT over a single-row spiral CT is a shorter acquisition time. Thus, the volume data set from a MD-CT is much less subject to motion or breathing artifacts. It is known from experience, that data from single-row spiral-CT are rather accurate as well, but with a view to the fact that comparative studies do not exist, and cannot be performed due to ethical considerations, one can only hypothesise that the findings are repeatable using single row technique. Volumetric analysis was not used, as it is very time-consuming and expensive [6] and its application has not yet become established. Furthermore, we did not use the body surface area (BSA), a well known influencing factor for kidney volume, [15-17], as the more common BMI was used as a criterion for the factor "obesity" [18], and the two indexes are not independent of one another. Moreover, apart from the fact, that the creatinine levels were normal in most of the patients, or < 2 mg/dl in a small subgroup of patients, respectively, nothing is known about possible correlations of kidney function and size measurements in the present study. Results from ultrasonographic volume measurements of kidneys are promising [19], but further research is necessary in order to improve these opportunities.

In principle, kidney length can be estimated using ultrasound, MRI, intravenous pyelograms and CT, amongst others [14]. The CT predicts kidney length better than other modalities, but all modalities are connected with prediction errors in view of the kidney length [14]. The existent CT data regarding the kidney length were anticipated to be improved considerably, because they stemmed from 7 mm thick slices [14], in the worst case bringing about an estimation error of as much as 14 mm in the z-axis due to partial volume artefacts. However, the consistency of the data from our study, compared to the study of Kang et al. [13] has to be regarded as good, and even the values of the standard deviation are in agreement. The values presented here are for kidney length are slightly higher, because the longitudinal axis was exactly adjusted in every single kidney in 3D, thus correcting the underestimation of length due to projection errors in the x and y axes. This underestimation of length is the main problem when using ultrasound as well, as can be inferred from the data of Kang et al. [14]. The longitudinal axis of a kidney is not always perfectly adjustable in ultrasound. Moreover, the ultrasound technique is dependent on the sonographer [20]. Only little is known about kidney size measurements using MRI. It seems to be better than ultrasound in terms of estimating the kidney length [21]. Though a very high agreement with data obtained from CT may be expected, a study comparing CT and MRI in this regard is currently missing.

The differentiated observation of kidney sizes is of great significance clinically, as many diseases are associated with changes in kidney size [4]. The normal range is large [22], and what is "normal" depends on many factors. Within the standard deviation of LPPs there are values <9 cm in slim older women and up to 13 cm for men in their fifties. In the presence of other factors such as normal ADRAs or conspicuous body size, there are cases where LPPs of <8 or >14 cm can be considered normal and not be misunderstood as a sign of a cirrhotic kidney or acute kidney failure. Non-gender-specific data, according to which normal right kidneys are 11 ± 1 cm and left kidneys 11.5 ± 1 cm long [23], or 11 and 12 cm long, 5 and 7 or 7.5 cm wide, and 2.5 or 3 cm thick [24,25], are not particularly useful in clinical practice. The influencing factors for size must be viewed individually to arrive at any relevant conclusions and information.

The age-related decrease of the LPP [4] and PW [26] are well known. The increase of the LPP in men up to their fifties has already been documented in data by Simon [27], who, however, did not consciously record it. While the LPP in men is only slightly larger than for women in the third decade, after the fifth decade it is about 10 mm longer, i.e. 10% of the LPP. We assume that sex hormones influence this. Be that as it may, with respect to the nonlinear relation of LPP to age in men, we waived the comparative gender-specific estimation of the influence of age based on various linear and nonlinear models, as it would have been of dubious value. It should be noted only that age is the greatest negative influencing factor on both CW and PW.

The great influence of BMI on LPP, CW, and PW was anticipated due to the known influence of body weight and BSA [6]. It appears to be more pronounced in women. The differences in the average values of clinically and morbidly obese patients in comparison with patients of normal weight [28-30] were as much as 20%.

The influence of height on LPP is also well documented [4] - it is by far the greatest independent predictor. Its influence on the PW is approximately comparable to that of BMI, but strangely enough, there is no influence on the CW. This could facilitate arriving at conclusions as to the extent of arteriosclerotic renal disease based on the CW [8].

The factor "stenosis of the renal arteries" is also well known [8]. The part it plays in the models with respect to the LPP is somewhat less pronounced than height, BMI, or gender. This is compatible with the observation that within a natural course of 33 months, only 16.2% of kidneys in patients with at least one renal artery stenosis are reduced in length by at least 1 cm - after exclusion of 7.7% of the kidneys in the patient group with either renal artery occlusion or atrophy [31]. Thus, less than 8% of the kidneys with arterial stenosis are found to be atrophic. Due probably to a partially collinear effect with age with respect to CW and PW, this factor ceased to be an independent predictor on one side of the models. Furthermore, the known independent and also age-related atrophogenic effect of arterial hypertension may also play a part [31].

The influence of the position of the kidneys on length is conspicuous - the further cranial and dorsal a kidney is, the longer it is. The former is known for the special case of pelvic kidneys [32]. We have no explanation for this strong effect, which surpasses even that of a renal artery stenosis. Since the length of the shortest perpendicular from the dorsal kidney surface to the dorsal fascia, as an indicator of perirenal fat, correlates positively with the thickness of the renal capsule and the BMI, the opposite was expected.

Duplex kidneys are longer than the contralateral kidneys [33]. They are supplied by ADRAs more frequently than "normal" kidneys [34]. The possible more likely persistence of vessels in relation to the length of the kidney is a plausible, although hypothetical explanation for the pronounced independent positive interrelation between LPP and the number of vessels.

Since kidneys do not increase in width in women, and in men get only slightly wider with increasing age, while the renal pelvis widens greatly in both men and women, the RPRP becomes accordingly smaller. It can therefore be assumed that renal tissue is replaced by fat. This would make the RPRP ultimately a measure for the atrophy of the organs, contrary to the currently valid opinion [4]. Whether and under what circumstances cortical or medullar tissue is replaced must be the subject of future research.

## Conclusion

In summary we can say that there is a wide range in kidney sizes. LPPs of less than 8 cm as well as more than 14 cm can be normal. Among the strong positive influencing factors on LPP, CW, and PW in adults, the BMI, male gender, and height are most important. Cranial and posterior position of the kidneys is also significant. ADRAs have a strong positive effect. With respect to the cortex, the absence of the contralateral kidney is by far the most important factor. The most important negative influencing factors are age and stenosis of the renal artery. Due to the complex interaction of the factors described, the sizes presented here should serve as a reference point for the difficult differentiation between what is normal and pathological in individual cases in clinical routine.

## Competing interests

The authors declare that they have no competing interests.

## Pre-publication history

The pre-publication history for this paper can be accessed here:

http://www.biomedcentral.com/1471-2490/9/19/prepub
